# Big Five Personality Traits and Trajectories of Fertility Expectations Across the Reproductive Age Period

**DOI:** 10.1111/jopy.70021

**Published:** 2025-09-10

**Authors:** İlayda Özoruç, Jeroen Vermunt, Katya Ivanova, Manon van Scheppingen

**Affiliations:** ^1^ Department of Developmental Psychology Tilburg University Tilburg the Netherlands; ^2^ Department of Sociology Tilburg University Tilburg the Netherlands; ^3^ Department of Methodology Tilburg University Tilburg the Netherlands

**Keywords:** Big Five personality traits, fertility expectations, joint latent class modeling, parenthood, reproductive age

## Abstract

**Objective:**

In recent decades, increased freedom of choice and advancements in fertility regulation have allowed individuals to follow different fertility paths. This greater autonomy provides room for personality traits to shape long‐term fertility expectations, which in turn can be predictive of fertility outcomes. The present study investigates how Big Five personality traits are related to fertility expectations trajectories and outcomes.

**Method:**

We used a representative Dutch sample who was non‐parents at the start of the study (*N* = 5231). We explored the development of fertility expectations (i.e., *Do you think you will have children in the future?*) across ages 18–49. We conducted joint latent class analyses.

**Results:**

Having a stable expectation to become a parent was the largest class. However, the majority deviated from this trajectory. The identified classes varied in their probability of becoming a parent or not. Men and women who had stable parenthood expectations scored higher on agreeableness and extraversion. Additionally, men scored higher on conscientiousness and openness, and lower on neuroticism compared to some of the other classes.

**Conclusions:**

People show substantial variation in their fertility expectation trajectories across reproductive age. Especially in men, Big Five personality traits are related to fertility expectations trajectories.

## 1 Introduction

Over the past 50 years, the worldwide average fertility rate has been decreasing and childlessness has become more prevalent (Frejka 2017; Miettinen et al. 2015; United Nations Department of Economic and Social Affairs, Population Division 2022). Increases in freedom of choice and the development of new fertility regulation technologies over the past decades have contributed to the ability of people to choose whether and, if so, when to become parents (Lesthaeghe 2010; Miettinen and Szalma 2014; Rybińska 2021). People can deviate from traditional parenthood transitions (e.g., becoming a parent around “an ideal age”) by postponing parenthood, choosing to remain childless, and changing their decision over time (Rybińska, 2021; Zaidi and Morgan 2017). Which pathway people choose is likely influenced by individual‐level characteristics, such as personality trait levels (Avison and Furnham 2015; Berg et al. 2013; Buhr and Huinink 2017; de Nijs et al. 2014; Hutteman et al. 2013; Jokela et al. 2009, 2011; Jokela 2012; Le Moglie et al. 2015; Mencarini et al. 2022).

While there is a substantial amount of research on fertility behavior (i.e., having children or not) and fertility intentions (i.e., trying to get pregnant in the short‐term or not; Hutteman et al. 2013; Jokela et al. 2009; Le Moglie et al. 2015), research on the development of fertility expectations (i.e., expecting to have children in the future or not) is limited (Gemmill 2019; Hayford 2009; Philipov and Bernardi 2012; Rybińska and Morgan 2019). It is important to examine expectations in addition to behavior and intentions because they reflect what individuals realistically anticipate for their reproductive future, even without concrete plans. Unlike intentions, which imply a commitment to act, expectations accommodate uncertainty and ambivalence. Fertility expectations serve as a valuable intermediate construct, bridging long‐term desires and actual behavior, and offer a more flexible lens on fertility decision‐making (Philipov and Bernardi 2012). This raises the following questions: What proportion of individuals maintain a consistent expectation of becoming a parent from an early age? Conversely, what proportion of individuals experience variations in this expectation, such as uncertainty or a lack of expectation to have children during young adulthood? In the present study, we answer these questions by studying the stability and change in fertility expectations in Dutch adults across the reproductive age period (i.e., 18–44 years old for women and 18–49 years old for men). Past literature on this topic has mainly focused on more specific subgroups (e.g., only women or only people who remained childless; Gemmill 2019; Hayford 2009). We built on this literature by using a representative sample and including all people of reproductive age (i.e., both people who eventually became parents and those who remained childless; both men and women).

Additionally, we contribute by exploring how various trajectories of fertility expectations differed in their probability of becoming a parent, and how the Big Five personality traits are related to each fertility expectation trajectory. One's personality might play a stronger role in predicting these longer‐term expectations compared to short‐term fertility behaviors and intentions (Fréchette et al. 2017), which are more strongly affected by current circumstances and limitations in one's life such as financial situation and partnership status (Fiori et al. 2017; Liefbroer 2009). Studying this association can help to refine theories on personality and fertility decisions and be a starting point for future research on mechanisms that may explain the different fertility expectations and outcomes in young adulthood.

### 1.1 Previous Studies on Trajectories of Fertility Expectations

In the literature, *fertility expectations* refer to “number of children that an individual thinks might be achieved under the restriction of prevalent conditions but independently of whether the children were intended or not” (Philipov and Bernardi 2012, 508). People differ in fertility expectations and can change or maintain these expectations (Hayford 2009). On the one hand, people can have a stable fertility expectation over time, especially people who expected either to be childless or to have two children in the future compared to people who expected a different number of children (Gemmill 2019; Hayford 2009). On the other hand, fertility expectations can also increase or decrease, with decreases being more prevalent as people age (Brauner‐Otto and Geist 2018; Gemmill 2019; Hayford 2009; Iacovou and Tavares 2011; Verweij et al. 2021; Yarger 2011). Moreover, some people can keep switching their expectations between becoming a parent and remaining childless which leads to an unstable fertility expectation pattern (Gemmill 2019; Hayford 2009). Worthy of note, some people might be explicitly uncertain about whether to have children or not in the future (Ní Bhrolcháin and Beaujouan 2015). While previous studies have yielded valuable insights, they have overlooked several critical aspects. The present study addresses three of these neglected areas.

First, most of the studies did not include individuals who are unsure whether they will have children or not in the future (Hayford 2009; Iacovou and Tavares 2011; Verweij et al. 2021). However, uncertainty is a rational, real and widespread response for fertility in developed societies where many alternatives are increasingly possible (Ní Bhrolcháin and Beaujouan 2015). A longitudinal study on fertility intentions has found that uncertainty acts as a transitional state between certainly intending and not certainly intending to have a child (Kuhnt et al. 2021). Therefore, taking uncertainty into account as a valid response option is necessary and informative. In the present study, we will dedicate attention to uncertainty by examining “I do not know” responses.

Second, previous studies have predominantly focused on pathways into childlessness and often overlooked the pathways of those who became parents (Buhr and Huinink 2017; Gemmill 2019; Verweij et al. 2021). This neglect has resulted in a significant gap in understanding the development of fertility expectations of people who pursue parenthood. In the present study, we will observe the development of fertility expectations of people who became a parent as well as those who did not by the end of the observation period.

Lastly, most of the previous studies neglected fertility expectations of men by solely focusing on women and thus did not present a comparison between the two genders (Gemmill 2019; Hayford 2009; Rybińska and Morgan 2019). The decrease in gendered parenthood norms has paved the way for more equal opportunities and responsibilities for men and women. Both are expected to participate in homemaking as well as breadwinning activities. As women have entered the labor market, men have been expected to take on more responsibilities within the family domain (Preisner et al. 2020). However, women still seem to take on more homemaking tasks, including taking care of children, and have a greater difficulty in balancing parenthood and career compared to men (Cerrato and Cifre 2018). Adding to these challenges, women have a more limited time window of natural reproductivity ability compared to men. For these reasons, women and men can show differences in fertility expectations. The present study therefore compares the development of fertility expectations of women and men.

In the present study, our first aim is to identify different developmental trajectories of fertility expectations in men and women living in the Netherlands across the reproductive age period. In the Netherlands, voluntary childlessness is increasingly socially acceptable (Noordhuizen et al. 2010) and the use of contraceptives is widespread (Marra et al. 2020). In such a context, we can possibly observe more variation in how people imagine their futures regarding having a child or not. We expect to find different developmental trajectories of fertility expectations. Based on the literature, we cannot indicate the number of likely trajectories. Therefore, we explore the number of trajectories and in what way the trajectories differ. Our second aim is examining how Big Five personality traits are related to different trajectories of fertility expectations.

### 1.2 Big Five Personality Traits and Development of Fertility Expectations

Some previous studies have attempted to explain individual differences in fertility decisions by mostly demographic, cultural, and economic factors (for a review, see Balbo et al. 2013). For example, being actively religious and living with a partner are associated with a higher desire for parenthood, whereas a negative financial situation has been related to greater fertility uncertainty (Craig et al. 2014; Dommermuth et al. 2011; Testa and Basten 2014). However, the expanded range of individual choices regarding fertility decisions could amplify the role of personal dispositions (Jokela 2012; Mencarini et al. 2022; Zaidi and Morgan 2017). Previous cross‐sectional and longitudinal studies have found mixed results for the association between personality and fertility behavior (Berg et al. 2013; de Nijs et al. 2014; Jokela et al. 2009, 2011; Jokela 2012; Le Moglie et al. 2015; Mencarini et al. 2022) and fertility intentions (Avison and Furnham 2015; Buhr and Huinink 2017; de Nijs et al. 2014; Hutteman et al. 2013; Pinquart et al. 2008). One explanation for the mixed results can be that fertility behaviors and intentions are restricted by ongoing circumstances and limitations in one's life (Fiori et al. 2017; Liefbroer 2009). These circumstances may restrict the impact of personality traits on fertility‐related decisions. In the present study, we explore whether personality traits show an influence on the development of longer‐term fertility expectations. Since we do not know what kind of trajectories we will identify, we do not present clear hypotheses on how personality traits will be related to each trajectory. However, we do have expectations on how each trait can be related to the initial state, stability, and certainty of the fertility expectations.

Agreeableness is a trait representing warmth, sympathy, and kindness. Individuals with high agreeableness have been shown to perceive parenthood as a positive experience (de Nijs et al. 2014). Supporting this, many studies have shown that higher levels of agreeableness are related to higher fertility (Avison and Furnham 2015; Berg et al. 2013; Jokela 2012; Jokela et al. 2011; Le Moglie et al. 2015; Lundberg 2009). In addition, individuals with high agreeableness seem to be less ambivalent about their fertility decisions (Pinquart et al. 2008). However, this has only been examined cross‐sectionally. Based on the previous findings and the facets of the trait, we anticipate that people high in agreeableness may be more likely to expect to be a parent initially and be more stable and certain in their fertility expectations over time.

Neuroticism reflects a general tendency for negative affect and less positive emotions and is the opposite of emotional stability. Studies have shown mixed results; some studies have found a positive association whereas others found no or a negative association with fertility (Berg et al. 2013; Jokela 2012; Jokela et al. 2011; Le Moglie et al. 2015; Lundberg 2009). Higher neuroticism has also been related to higher ambivalence in fertility decisions (Pinquart et al. 2008). Based on the previous findings and the facets of the trait, we anticipate that people high in neuroticism may be more likely to be unstable and uncertain in their fertility expectations over time regardless of their initial expectation.

Openness to experience involves characteristics such as creativity and curiosity. Previous research has consistently shown that higher levels of openness are related to lower intentions to have a child and lower fertility rates, especially for women (Avison and Furnham 2015; Berg et al. 2013; Jokela 2012; Jokela et al. 2011; Le Moglie et al. 2015; Lundberg 2009; van Scheppingen et al. 2016). However, people high in openness might be open to the experience of becoming a parent as well as to the alternative activities that would be restricted in the case of parenthood (e.g., spontaneous outings, hobbies, traveling). They are more likely to postpone parenthood compared to those low in openness (Tavares 2010). This postponement can potentially lead to involuntary childlessness (Fiori et al. 2017). Based on the previous findings and the facets of the trait, we anticipate that people high in openness may be less likely to expect to be a parent and more likely to be unstable and uncertain in their fertility expectations over time.

Conscientiousness is a trait related to being responsible, careful, and planned. Some studies have shown that it is related to lower fertility intentions and lower fertility, especially for women (Jokela 2012; Jokela et al. 2011; Le Moglie et al. 2015). However, other studies have shown that it is related to higher fertility (Berg et al. 2013; Roberts and Bogg 2004). The direction of the effect might be related to differences in individual goals and values regarding family life and career (Jokela et al. 2011). For individuals prioritizing their careers, high conscientiousness might be linked to lower fertility intentions, whereas the opposite effect may be observed for those prioritizing family life. Based on the previous findings and the facets of the trait, we anticipate that people high in conscientiousness may be more likely to be stable and certain in their fertility expectations over time regardless of whether they expect to be a parent or not.

Extraversion refers to the tendency to be more outgoing, sociable, and energized by social interactions. Many studies have shown that high levels of extraversion are related to higher fertility (Avison and Furnham 2015; Berg et al. 2013; Jokela 2012; Jokela et al. 2011; Lundberg 2009). An explanation can be that people scoring high on extraversion have a higher propensity to find a partner, which in turn increases the likelihood of becoming a parent (Nettle 2005). Based on the previous findings and the facets of the trait, we anticipate that people high in extraversion may be more likely to expect to be a parent. We will explore how it is related to changes in fertility expectations over time.

## 2 Methods

### 2.1 Participants

The present study used data from the LISS panel (Longitudinal Internet studies for the Social Sciences; https://www.lissdata.nl). The LISS is an ongoing panel study that has started in 2007. It includes 5000 households, comprising nearly 7500 individuals. The panel consists of a true probability sample of households chosen from the population register by Statistics Netherlands (for more information, see Scherpenzeel 2011). We have applied several inclusion and exclusion criteria. First, we selected participants who completed the question about fertility expectations at least once between 2008 and 2022. Second, we restricted the age range to 18–44 years for women and 18–49 years for men to capture the reproductive age period during adulthood. Moreover, we included both men and women but excluded people who indicated their gender as “*other*” because this option was only available for 2022 and there were not enough participants (*n* = 20) to create a third gender group. We also excluded people who indicated that they had child(ren) when they entered the panel because the present study focuses on expectations on transitioning to parenthood rather than expectations on additional children.

The initial sample in the Family and Household study consisted of 7110 individuals in 2008. The attrition rate in the LISS panel is nearly 10% each year (Centerdata 2024). Therefore, refreshment samples have been added regularly to correct for any deviations in representativeness of the Dutch population (Scherpenzeel 2011; Leenheer and Scherpenzeel 2013). In total, 9074 participants reported on their fertility expectations at least once between 2008 and 2022. After applying our inclusion and exclusion criteria, the sample size decreased to 5231^1^. The average number of waves per participant was 3.60 (SD = 3.08) ranging from 1–15 waves. The majority of the participants (67.5%) responded to more than one wave (*n* = 1699 participated only once). The sample consisted of 52.1% women (*M*_age_ = 25.67, SD_age_ = 7.21) and 47.9% men (*M*_age_ = 28.00, SD_age_ = 8.75). During the data collection, 14.62% of the participants (*n* = 765) became parents for the first time, with the overwhelming majority (*n* = 668) being biological parents (see Table 1 for more details on participants background).

**TABLE 1:** Participant background variables.

Measure	Category	Percentage	*n*
Background	Dutch	65.6	3433
	Second generation foreign, non‐Western background	6.2	325
	Second generation foreign, Western background	5.6	295
	First generation foreign, non‐Western background	4.1	215
	First generation foreign, Western background	3.3	174
	NA	15.1	789
Education	Higher vocational education	24.5	1279
	Intermediate vocational education	23.9	1251
	Higher professional education, university education	20.2	1056
	Higher secondary education or preparatory university education	16.0	835
	Intermediate secondary education	9.6	503
	Primary school	2.6	138
	No completed education	1.9	100
	Other	0.9	49
	NA	0.4	20
Partner status	Ever‐partnered	66.8	3494
	Never‐partnered	31.4	1640
	NA	1.9	97
First time parenthood	Biological parent	87.3	668
	Step parent	7.9	58
	Foster parent	0.9	7
	Adoptive parent	0.3	2
	NA	3.9	30

*Note:* Education refers to the highest level of education achieved. See the Dutch equivalents: higher vocational education = hoger beroepsonderwijs, intermediate vocational education = middelbaar beroepsonderwijs in Dutch, higher professional education, university education = wetenschappelijk onderwijs, higher secondary education or preparatory university education = hoger algemeen voortgezet onderwijs/voorbereidend wetenschappelijk onderwijs, intermediate secondary education = voorbereidend middelbaar beroepsonderwijs, primary school = basisschool. Ever‐parented status refers to having a partner at least one measurement occasion.

Abbreviation: NA, Not Available.

### 2.2 Measures

#### 2.2.1 Fertility Expectations

Respondents reported on their fertility expectations at each annual time point (2008–2022) in the Family and Household questionnaire which is part of the LISS core study. The item was “*Do you think you will have children in the future?*” The response options were “*Yes*”, “*No*”, and “*I don't know*”.

#### 2.2.2 Big Five Personality Traits

The 50‐item International Personality Item Pool (Goldberg 1992) was used to measure the Big Five personality traits at each time point (2008–2022, except 2016). We used the first available response for each participant to represent the initial personality trait levels (see Table 2 for the distribution of the waves in which the first response was measured). Participants were asked to use a rating scale to assess how accurately each statement described themselves in relation to other people of the same sex and roughly the same age as they were. There were ten items for each trait. Sample items were: “*Feel comfortable around people*” for extraversion, “*Sympathize with others' feelings*” for agreeableness, “*Change my mood a lot*” for neuroticism, “*Follow a schedule*” for conscientiousness and “*Have a vivid imagination*” for openness. Response options were “*1 = very inaccurate, 2 = moderately inaccurate, 3 = neither inaccurate nor accurate, 4 = moderately accurate, 5 = very accurate*”. Cronbach's alphas were 0.74 for openness, 0.79 for conscientiousness, 0.88 for extraversion, 0.81 for agreeableness and 0.88 for neuroticism indicating an overall good level of internal consistency.

**TABLE 2:** Descriptive statistics for fertility expectations and parenthood across measurement waves.

Wave	*n*	Fertility expectations			First time parenthood	First available personality scores
		Yes	No	Unsure		
2008	1603	870	300	433	NA	1614
2009	1297	664	241	392	58	198
2010	1383	750	232	401	16	382
2011	1186	632	197	357	64	106
2012	1266	674	231	361	61	302
2013	1170	626	211	333	76	116
2014	1533	749	197	355	60	597
2015	1301	576	165	314	86	127
2016	1055	767	225	397	77	NA
2017	1389	640	182	334	53	384
2018	1156	545	164	305	42	227
2019	1014	716	216	377	46	89
2020	1309	716	216	377	32	342
2021	1002	495	194	313	60	114
2022	1096	547	220	329	34	181

*Note:* N_total_ = 5231. N_personality_ = 4779.

Abbreviation: NA, Not Applicable.

#### 2.2.3 Parenthood Status

Respondents reported on their parenthood status at each time point (2008–2022). It captured whether the participant had any child, including biological children (conceived with their partner or someone else), stepchildren, adoptive children, foster children, and deceased children. Response options were “*yes*” and “*no*”.

### 2.3 Procedure

During the recruitment of the panel, computer and internet connection were provided for households that needed it. Participants received an incentive of 5 euros prepaid and a second incentive of 10 euros if they got registered in the LISS panel. Individuals who accepted to participate received a confirmation email, and a letter with login code. Respondents provided informed consent for the use of the collected data in scientific and policy‐relevant research in compliance with the European General Data Protection Regulation (GDPR). The present study is approved by the Ethics Review Board of Tilburg University. The LISS Data Archive is free to use for researchers if data will be used for scientific, policy or socially relevant (i.e., non‐commercial) research. Researchers sign a statement to ensure the privacy and confidentiality of participants stating that information pertaining to individual persons and households will not be disclosed to any external parties. Researchers do not have access to any participants' identifying information such as name and address.

### 2.4 Data Analysis

We used IBM SPSS Statistics (Version 28) for data preparation and descriptive statistics. We conducted a joint latent class analysis to estimate the trajectories of fertility expectations prior to the birth of the first child using Latent GOLD 6.0 software (Vermunt and Magidson 2021). Based on the previous research, Nylund‐Gibson and Choi (2018) suggested that a sample size of 300–1000 is usually sufficient for latent class analyses and mixture models to function adequately. In the present study, we have larger samples (*n* = 2507 for men and *n* = 2724 for women) that ensure a sufficient power to run the planned analyses and draw conclusions.

#### 2.4.1 Step 1: Sample Inclusion and Missing Data

We included childless people from their entry into the panel until they became parents or until they did not respond anymore due to age restrictions, the end of observation period (2022), or drop out. Missingness after birth of the first child clearly cannot be assumed to be independent of fertility expectations. In order to deal with this issue, we modeled the (discrete time) hazard rate of first child simultaneously with the fertility expectations. Latent classes were allowed to differ not only with respect to the fertility trajectories, but also with respect to the probability of having a first child at a certain age (conditional on not having children yet). For this purpose, a binary logistic regression was specified for the variable “first child” with age as predictor. Such an approach for the simultaneous analysis of a longitudinal outcome and a time‐to‐event variable is usually referred to as joint modeling (Proust‐Lima et al. 2014). The joint latent class model was estimated with all the observed data available (for a detailed explanation of missing data, see preregistration: https://osf.io/enbvs/?view_only=c7a474398e3f4fc7b67c3c61de46a4c7; for a detailed explanation of how Latent GOLD handles missing data, see: Vermunt and Magidson 2016).

#### 2.4.2 Step 2: The Joint Latent Class Model

The core of the joint latent class model consisted of regression models for each of the two outcome variables of interest (i.e., fertility expectations and first‐birth hazard rate at a certain age). For fertility expectations and first‐birth hazard we used a nominal and a binary logistic regression model, respectively. To capture possible non‐linear changes in fertility expectations across the reproductive age period, we included various age variables as predictors. Specifically, we built a non‐linear model allowing a cubic age effect by using the B‐spline specification in Latent GOLD (Francis et al. 2016; Vermunt and Magidson 2021). Additionally, due to the broad age range, we specified one knot in the model, which allowed for a change in the cubic age effect and made the model more flexible (see Francis et al. 2016 for more detailed information about use of B‐splines and knots).

#### 2.4.3 Step 3: Model Selection

Next, we estimated models with an increasing number of classes, using a sufficiently large number of starting values to avoid local maxima. We determined the optimal number of classes by the lower Bayesian Information Criterion (BIC), which applies a stronger penalty for model complexity compared to other selection tools like the Akaike Information Criterion (AIC; Lin et al. 2002; van De Schoot et al. 2017). As additional comparison criteria, we considered the AIC, the AIC3 (Corrected AIC with penalty factor of 3) values, and a significant bootstrap likelihood‐ratio test (BLRT) using an alpha level of 0.01 (Nylund et al. 2007; van De Schoot et al. 2017; Vermunt 2024; Vermunt and Magidson 2021). Furthermore, we interpreted the longitudinal bivariate residuals which quantify whether the overall associations and the associations between consecutive time points (autocorrelations) are effectively captured by the specified model. A lower value suggests that there is a closer match between observed and estimated associations and the model indicates an adequate representation of the time‐based variations of the data. Lastly, we took additional factors into account such as classes being theoretically meaningful, avoiding trajectories that were too similar to each other, and a minimum class size of 5% of the sample size to justify a trajectory as a separate class. We also examined whether increasing the number of knots in the model affected the decision regarding the optimal number of classes, based on model fit values.

#### 2.4.4 Step 4: Personality and Class Membership

After deciding on the optimal number of classes, we examined whether personality differences were linked to different developmental trajectories of fertility expectations across reproductive age period in adulthood. Specifically, we examined whether the Big Five personality traits were associated with latent class membership using a biased adjusted 3‐step approach which takes misclassification into account in model building (for more details about the approach see: Vermunt 2010).

## 3 Results

Table 2 shows descriptive statistics across measurement waves. For each wave, there were between 1002 and 1603 participants. The most reported expectation was expecting to be a parent followed by being unsure and not expecting to be a parent, respectively. The majority of the Big Five personality traits scores were retrieved from 2008, representing the most available initial point.

### 3.1 Fertility Expectation Trajectories for Men

For men, the joint latent class analysis showed that a 5‐class model performed best based on the lowest BIC value compared to the models with a lower and higher number of classes, with acceptable class sizes (proportion of individuals in each class: 0.44, 0.19, 0.17, 0.12, 0.08, respectively), decreased autocorrelations, as well as clearly different and interpretable trajectories. Table 3 shows the model fit statistics for the models with 1 to 6 classes (see Table S1 for regression parameters). Increasing the number of knots showed no improvement in BIC values nor any significant additional information presented in the identified classes (see Table S2).

**TABLE 3:** Model fit statistics for the 1–6 class models for men.

Number of classes	BIC	AIC	AIC3	VLMR	BLRT	BVR (Lag1‐Lag2)	Entropy R^2^	Class proportions
1‐class	17816.49	17729.09	17744.09	—		287.56 241.48	1.00	1.0
2‐class	15014.93	14834.29	14865.29	2926.80 (*p* < 0.001)	< 0.001	69.85 46.87	0.688	0.61/0.39
3‐class	14429.82	14155.96	14202.96	710.34 (*p* < 0.001)	< 0.001	37.79 24.44	0.637	0.58/0.28/0.13
4‐class	14252.71	13885.62	13948.62	302.34 (*p* < 0.001)	< 0.001	24.70 14.83	0.533	0.49/0.22/0.19/0.10
5‐class	14162.78	13702.46	13781.46	215.15 (*p* < 0.001)	< 0.001	16.39 9.98	0.503	0.44/0.19/0.17/0.12/0.08
6‐class	14188.59	13635.04	13730.04	99.43 (*p* = 0.001)	< 0.001	12.85 7.62	0.460	0.34/0.20/0.16/0.15/0.09/0.06

*Note:* Number of starting values: sets = 500, iterations = 1000 for men.

Abbreviations: AIC, Akaike information criterion; BIC, Bayesian information criterion; BLRT, bootstrap likelihood‐ratio test; BVR (Lag1‐Lag2), Longitudinal bivariate residuals for fertility expectations; VLMR, Vuong‐Lo‐Mendel‐Rubin test.

**FIGURE 1:**
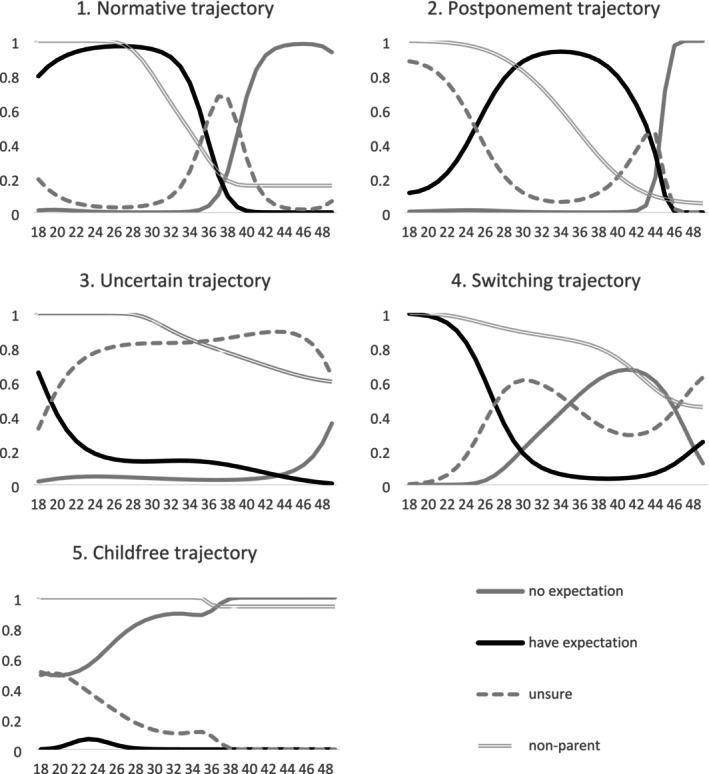
Fertility expectations trajectories of men identified in the 5 class model. This figure displays five distinct trajectories of fertility expectations among men. Each trajectory represents a different pattern of expectations across age. X‐axis: Age in years. Y‐axis: Proportion of individuals in each class expressing a given expectation. Class proportions: 0.44/0.19/0.17/0.12/0.08.

Figure 1 shows the identified five trajectories for men. Results indicated that men belonging to the first class (44%) had expectations at the age of 18 to have a child in the future and kept this expectation until their mid‐thirties. Between the age of 30 and 40, an increasing proportion of the men switched to being unsure. Then, there was a steep increase in men expecting no children, who kept this expectation until the end of the observation period. Eighty‐four percent of the men in this class became a parent (Median age ≈33 years old). We named this class the *normative* trajectory. Most men belonging to the second class (19%) were unsure about their expectations at the age of 18 years old but then the majority of the sample slowly switched to expecting to have a child from that age and on. From age 25, most men were expecting a child, and this expectation was stable until the age of 40 where expectations drastically changed to not expecting a child. Ninety‐four percent of the men in this class became parents (Median age ≈35 years old). We named this class the *postponement* trajectory. Most of the men belonging the third class (17%) had expectations at the age of 18 to have a child but most of them switched to being unsure about their expectation in their early twenties and maintained this uncertainty state throughout their reproductive period. Thirty‐nine percent of the men in this class transitioned to parenthood (Median age ≈36 years old). We named this class the *uncertain* trajectory. Men belonging to the fourth class (12%) had expectations at the age of 18 to have a child in the future. However, by their mid‐twenties, most kept switching between expecting no children and being unsure throughout their reproductive period. Fifty‐four percent of the men in this class became parents (Median age ≈40). We named this class the *switching* trajectory. Finally, men belonging the fifth class (8%) were divided between being unsure and expecting no children at the age of 18. During their twenties and onwards, most men switched to expecting no children and kept this expectation stable throughout their reproductive period. Only 6% of the men in this class transitioned to parenthood (Median age ≈36 years old). We named this class the *childfree* trajectory.

### 3.2 Fertility Expectation Trajectories for Women

For women, the joint latent class analysis showed that a 6‐class model performed best based on the lowest BIC value compared to the models with a lower and higher number of classes, with acceptable class sizes (0.40, 0.15, 0.14, 0.12, 0.12, 0.7 proportion of individuals in each class, respectively), decreased autocorrelations, as well as clearly different and interpretable trajectories. Table 4 shows the model fit statistics for the models with 1–7 classes (see Table S3 for regression parameters of 6 class model). Increasing the number of knots showed no improvement in BIC values nor any significant additional information presented in the identified classes (see Table S4).

**TABLE 4:** Model fit statistics for the 1–7 class models for women.

Number of classes	BIC	AIC	AIC3	VLMR	BLRT	BVR (Lag1‐Lag2)	Entropy *R* ^2^	Class proportions
1‐class	18160.28	18071.64	18086.64	—		289.25 222.22	1.000	1.0
2‐class	15356.11	15172.91	15203.91	2930.73 (*p* < 0.001)	< 0.001	74.62 47.62	0.683	0.68/0.32
3‐class	14832.06	14554.29	14601.29	650.61 (*p* < 0.001)	< 0.001	44.58 22.09	0.632	0.62/0.28/0.10
4‐class	14681.29	14308.96	14371.96	277.33 (*p* < 0.001)	< 0.001	31.18 14, 95	0.552	0.48/0.30/0.13/0.09
5‐class	14622.92	14156.05	14235.05	184.92 (*p* < 0.001)	< 0.001	21.14 9.83	0.511	0.46/0.20/0.13/0.12/0.09
6‐class	14614.43	14052.99	14147.99	135.05 (*p* < 0.001)	< 0.001	14.34 5.58	0.465	0.40/0.15/0.14/0.12/0.12/0.07
7‐class	14651.93	13995.94	14106.94	89.05 (*p* = 0.002)	< 0.001	13.28 6.21	0.459	0.38/0.14/0.14/0.13/0.12/0.05/0.04

*Note:* Number of starting values: sets = 750, iterations = 1500 for women.

Abbreviations: AIC, Akaike information criterion; BIC, Bayesian information criterion; BLRT, bootstrap likelihood‐ratio test; BVR (Lag1‐Lag2), Longitudinal bivariate residuals for fertility expectations; VLMR, Vuong‐Lo‐Mendel‐Rubin test.

**FIGURE 2:**
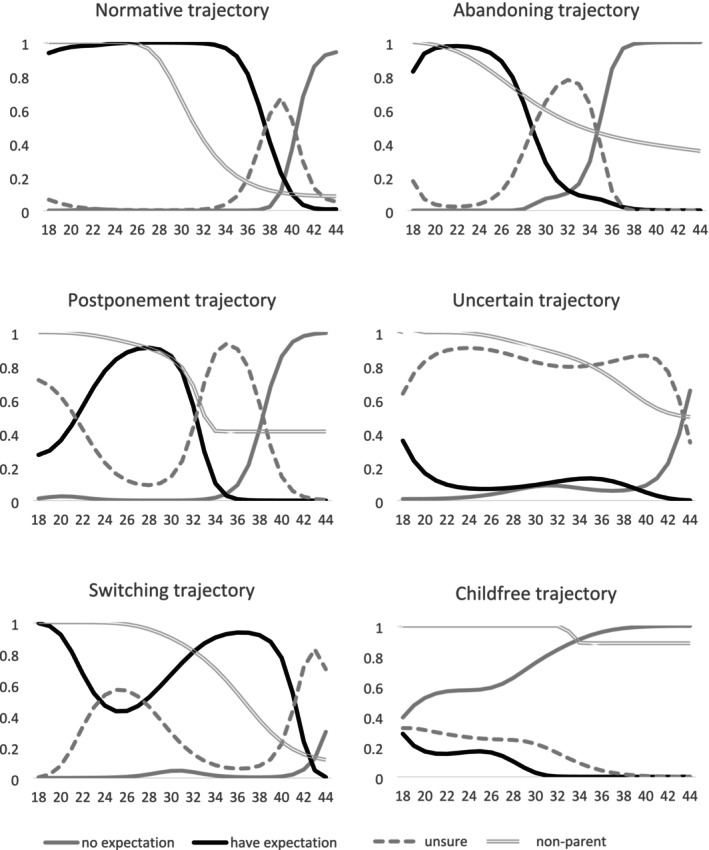
Fertility expectations trajectories of women identified in 6 class model. This figure displays six distinct trajectories of fertility expectations among women. Each trajectory represents a different pattern of expectations across age. X‐axis: Age in years. Y‐axis: Proportion of individuals in each class expressing a given expectation. Class proportions: 0.40/0.15/0.14/0.12/0.12/0.07.

Figure 2 shows the trajectories for each of the six classes for women. Very similar to the normative trajectory of men, women belonging to the first class (40%) had expectations at the age of 18 to have a child in the future and kept this expectation stable until their mid‐thirties, where a peak of being unsure began, followed by a drastic switch to most individuals expecting no children through their forties. Ninety‐two percent of the women in this class became parents (Median age ≈31 years old). We named this class the *normative* trajectory. In addition to the trajectories observed in men, 15% of the women showed an additional trajectory similar to the normative trajectory, in which women switched to the unsure state earlier, around the age of 25, and changed to not expecting a child around their mid‐thirties. Sixty‐five percent of the women in this class became parents (Median age ≈28 years old). Most of these women became parents while they were either unsure or did not expect to have a child and thus had already abandoned the expectation of becoming a parent. We named this class the *abandoning* trajectory. Most of the women belonging to the third class (14%) were unsure about their expectations at the start, but then the majority slowly switched to expecting to have a child and remained stable in this expectation across their twenties. Unlike the postponement trajectory of men, most women started to switch back to being unsure during their late twenties and switched from being unsure to not expecting any children during their late thirties, where they maintained this expectation for the rest of their reproductive period. Fifty‐nine percent of the women in this class became parents (Median age ≈32 years old). We named this class the *postponement* trajectory. Very similar to the uncertain trajectory of men, women belonging to the fourth class (12%) were mostly unsure about their expectation throughout their reproductive age period. Fifty percent of the women in this class transitioned to parenthood (Median age ≈36 years old). We named this class the *uncertain* trajectory. Women belonging to the fifth class (12%) had expectations at the age of 18 to have a child in the future, but half of the women slowly switched to being unsure. After their mid‐twenties, most switched back to expecting to have children and kept it stable until their forties, where the rise of being unsure and expecting no children started. Eighty‐eight percent of the women in this class transitioned to parenthood (Median age ≈36 years old). We named this class the *switching* trajectory, which had a different switching pattern compared to the switching trajectory of men. Finally, women belonging to the sixth class (7%) were divided between the three expectation options at the beginning. By their mid‐twenties, most people switched to not expecting any children and maintained this expectation across their reproductive age period, similar to the childfree trajectory of men. Twelve percent of the women in this class transitioned to parenthood (Median age ≈33 years old). We named this class the *childfree* trajectory.

### 3.3 Big Five Personality Traits and the Fertility Expectation Trajectories

After determining the number of classes, we applied the biased adjusted 3‐step approach separately for men and women to examine how the trajectories differed on the Big Five personality traits. Big Five personality traits scores were available for 91.4% of the participants (*n* = 4779).

**FIGURE 3:**
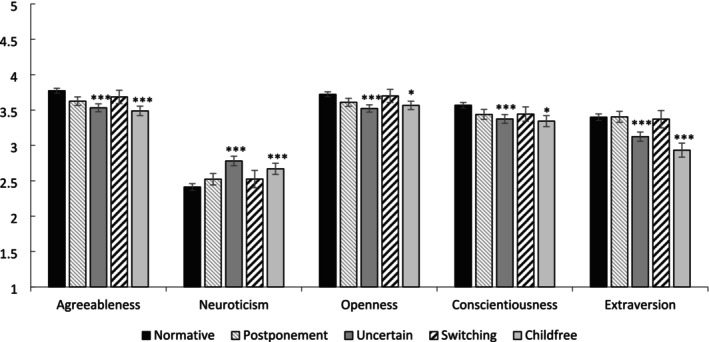
Fertility expectations trajectories and Big Five personality trait levels of men. *p < 0.05, **p < 0.01, ***p < 0.001 indicating a significant difference compared to the normative trajectory. Error bars show standard errors. Overall mean levels for men: Agreeableness (*M* = 3.67, SD = 0.68), Neuroticism (*M* = 2.53, SD = 0.87), Openness (*M* = 3.65, SD = 0.63), Conscientiousness (*M* = 3.48, SD = 0.76), Extraversion (*M* = 3.31, SD = 0.88).

For men, results showed significant small differences in agreeableness (*R* ^2^ = 0.040), neuroticism (*R* ^2^ = 0.042), openness (*R* ^2^ = 0.025), conscientiousness (*R* ^2^ = 0.021), and extraversion (*R* ^2^ = 0.049) across the classes. Taking the normative class as the reference group, paired comparisons indicated that people in the uncertain and childfree classes had lower levels of agreeableness, higher levels of neuroticism, lower levels of openness, lower levels of conscientiousness, and lower levels of extraversion (see Figure 3 for visualization of mean levels of the traits across the classes and Table 5 for details, including *p*‐values and Cohen's *d* values). Results were mostly in line with our expectations; however, we found higher levels of openness in the normative class instead of the expected lower levels compared to the uncertain and childfree classes.

**TABLE 5:** Big five personality trait level differences comparing the classes of men.

	1 vs. 2			1 vs. 3			1 vs. 4			1 vs. 5		
	Wald	*p*	*d*	Wald	*p*	*d*	Wald	*p*	*d*	Wald	*p*	*d*
Agreeableness	3.46	0.063	0.29	13.64	< 0.001	0.47	0.57	0.450	0.17	14.24	< 0.001	0.55
Neuroticism	1.02	0.310	−0.17	20.63	< 0.001	−0.56	0.55	0.460	−0.17	8.03	0.005	−0.39
Openness	2.09	0.150	0.23	10.53	0.001	0.41	0.04	0.840	0.05	5.10	0.024	0.32
Conscientiousness	1.88	0.170	0.22	6.93	0.009	0.33	1.01	0.310	0.21	6.49	0.011	0.39
Extraversion	0.00	0.960	−0.01	12.23	< 0.001	0.41	0.04	0.850	0.04	18.01	< 0.001	0.70

*Note:* The reference class was the Normative class. 1 vs. 2 = Normative vs. Postponement. 1 vs. 3 = Normative vs. Uncertain. 1 vs. 4 = Normative vs. Switching. 1 vs. 5 = Normative vs. Childfree. *d* = Cohen's *d*. Cohen's *d* is calculated by dividing the difference in mean levels of the traits between the groups by the pooled standard deviation of the overall men sample.

**FIGURE 4:**
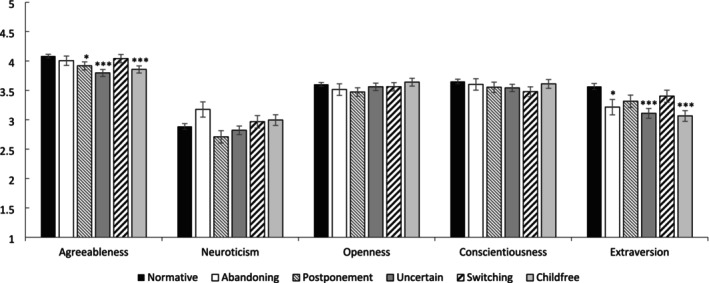
Fertility expectations trajectories and Big Five personality trait levels of women. *p < 0.05, **p < 0.01, ***p < 0.001 indicating a significant difference compared to the normative trajectory. Error bars show Standard Errors. Overall mean levels for women: Agreeableness (*M* = 3.99, SD = 0.59), Neuroticism (*M* = 2.91, SD = 0.92), Openness (*M* = 3.56, SD = 0.63), Conscientiousness (*M* = 3.59, SD = 0.72), Extraversion (*M* = 3.37, SD = 0.92).

For women, results suggested that agreeableness (*R* ^2^ = 0.047) and extraversion (*R* ^2^ = 0.070) showed significant small differences across the classes. Taking the normative class as the reference group, paired comparisons showed that women in the uncertain, postponement, and childfree classes had lower levels of agreeableness, like men. Moreover, women in the uncertain, abandoning, and childfree classes had lower levels of extraversion, similar to men. Unlike men, neuroticism, openness, and conscientiousness levels did not differ significantly between the classes for women (see Figure 4 for visualization of mean levels of the traits across the classes and Table 6 for details, including *p*‐values and Cohen's *d* values). Findings related to extraversion and agreeableness were mostly in line with our expectations; however, our expectations regarding neuroticism, openness, and conscientiousness were not supported.

**TABLE 6:** Big five personality trait level differences comparing the classes of women.

	1 vs. 2			1 vs. 3			1 vs. 4			1 vs. 5			1 vs. 6		
	Wald	*p*	*d*	Wald	*p*	*d*	Wald	*p*	*d*	Wald	*p*	*d*	Wald	*p*	*d*
Agreeableness	0.66	0.420	0.17	3.96	0.047	0.36	20.67	< 0.001	0.45	0.24	0.630	0.09	11.58	0.001	0.46
Neuroticism	3.48	0.062	−0.44	1.94	0.160	0.27	0.65	0.420	0.44	0.38	0.540	−0.12	1.17	0.280	0.43
Openness	0.56	0.460	0.18	2.22	0.140	0.27	0.38	0.540	0.45	0.20	0.660	0.08	0.29	0.590	0.46
Conscientiousness	0.17	0.680	0.09	0.83	0.360	0.17	2.55	0.110	0.46	2.78	0.095	0.31	0.23	0.630	0.45
Extraversion	4.91	0.027	0.52	3.79	0.052	0.37	25.22	< 0.001	0.42	1.61	0.200	0.24	23.70	< 0.001	0.43

*Note:* 1 vs. 2 = Normative vs. Abandoning. 1 vs. 3 = Normative vs. Postponement. 1 vs. 4 = Normative vs. Uncertain. 1 vs. 5 = Normative vs. Switching. 1 vs. 6 = Normative vs. Childfree. *d* = Cohen's *d*. Cohen's *d* is calculated by dividing the difference in mean levels of the traits between the groups by the pooled standard deviation of the overall women sample.

## 4 Discussion

In the present study, we focused on the development of long‐term fertility expectations, which sets us apart from previous studies that mostly concerned short‐term fertility intentions or behaviors. We aimed to identify different developmental trajectories of fertility expectations in reproductively aged men and women living in the Netherlands. As expected, we found different trajectories of fertility expectations. Joint latent class analysis identified five classes for men and six classes for women. We named them the *normative, postponement*, *uncertain*, *switching*, *childfree* trajectories for both genders, and additionally identified the *abandoning* trajectory specifically for women. Across the classes, the models estimated roughly 68% of men and 72% of women transitioning to parenthood across their reproductive age period. Our second aim was to explore how the Big Five personality traits were related to these different expectations trajectories. For men, we found differences between the classes on all Big Five traits, whereas only two traits were related to the trajectories of women. Men and women in the normative class scored higher on agreeableness and extraversion; additionally, men, but not women, showed higher levels of conscientiousness and openness, and lower levels of neuroticism compared to the other classes, mostly to the uncertain and childfree classes.

### 4.1 Identified Fertility Expectation Trajectories

We found that the normative class was the most prevalent class compared to the other identified classes for both men (44%) and women (40%). This trajectory is in line with what Dutch people reported to be the ideal timing of parenthood. That is, Dutch people report the ideal age of parenthood being between 27 and 29 years old and the upper age of parenthood being between 42 and 47 years old (see Billari et al. 2021). In line with our findings, previous research has shown that people commonly shift away from expecting to be a parent when coming closer to the end of their reproductive age (Brauner‐Otto and Geist 2018; Gemmill 2019; Hayford 2009; Iacovou and Tavares 2011; Verweij et al. 2021; Yarger 2011). The overwhelming majority of men and women in the normative class became parents. This is aligned with the previous research that found fertility intentions are strong indicators of the likelihood of becoming a parent, especially when the intentions are more certain (Schoen et al. 1999). Even though the normative class was the biggest class, the majority of the men and women followed different paths in their fertility expectations.

We found that a small but substantial group of men (8%) and women (7%) expected to be childless starting from the age of 18 and maintained this expectation for the rest of their reproductive age period (i.e., the childfree trajectory). Some people might already know from an earlier age that it will be difficult for them to become a parent (e.g., because of physical or mental health problems). Yet, it is likely that many people in this class voluntarily opted out of parenthood (i.e., childfree; Blackstone and Stewart 2012). This corresponds with the previously found rates of voluntary childlessness (average of 10%) in the Netherlands (Miettinen et al. 2015). Notably, this trajectory was the only trajectory where a majority showed no expectation to have children starting from the age of 18 and remained childless. This supports the previous childlessness research which also found that childfree people tend to be persistent early deciders (Gemmill 2019; Neal and Neal 2023). Our findings highlight that an early declaration of not expecting to have a child can be seen as a predictive response for future childlessness. This observed persistence of childfree expectations from early adulthood for approximately 7% to 8% of individuals highlights the importance of recognizing non‐parenthood as a legitimate and stable life course. Moreover, our findings on personality trait differences between this group and individuals following the normative trajectory enrich the understanding of fertility decline by emphasizing the role of personal agency, alongside the economic and structural factors typically emphasized in policy discussions.

We make several contributions with the present study. Our first contribution is modeling the fertility expectations of people who become parents as well as those who remain childless. Similar to previous research on fertility intentions and expectations (Buhr and Huinink 2017; Gemmill 2019; Verweij et al. 2021), we found that there are different trajectories that can result in being childless. Similarly, we showed that there are different paths of fertility expectations that can lead to parenthood. This supports that fertility expectations and decisions are a long‐term process rather than a preset goal, and uncertainty and changes are parts of this process (Ní Bhrolcháin and Beaujouan 2015). Both parents and childless people should not be treated as homogenous groups in future research.

Our second important contribution is including people who were uncertain regarding their fertility expectations. Consequently, we found a group of men (17%) and women (12%) who were constantly unsure regarding their expectation of having a child or not throughout their reproductive age period (i.e., the uncertain trajectory). In addition to this, 60% of men and 50% of women in this class remained childless. This is the second highest number of childlessness observed in the identified classes, the first being the childfree trajectory. Previous studies defined uncertainty as a transitional state between expecting and not expecting to have a child (Jones 2017; Kuhnt et al. 2021). We showed that being constantly unsure about parenthood is also possible and not uncommon. This indicates that uncertainty about fertility expectations is a meaningful response and can be a long‐term state (Ní Bhrolcháin and Beaujouan 2015).

Our third contribution is comparing trajectories of fertility expectation of men and women. Most of the identified trajectories were similar across gender, but there were also important differences. Firstly, early abandonment of the expectation of becoming a parent was only observed in women (i.e., the abandoning trajectory). Moreover, women in the postponement class showed an earlier shift to being unsure and then to not expecting any children, whereas men maintained their expectation to become a parent for a longer period of time. There can be several reasons for this difference. Even though relaxed gendered parenthood norms allow women to pursue a career and motherhood at the same time (Goldscheider et al. 2015), women still experience more difficulty in balancing both family and work life than men (Cerrato and Cifre 2018). In addition, despite the improved parental leave policies in the Netherlands, many women may consider the career costs of children and might need to make a choice between their career and parenthood, while men seem to be not affected in the same way (Adda et al. 2017). Lastly, the fact that increased age has a bigger effect on infertility and adverse fertility outcomes for women compared to men might explain these earlier shifts and the differences in the percentage of parents in these trajectories (Dunson et al. 2004; Schmidt et al. 2012).

### 4.2 Big Five Personality Traits and the Identified Fertility Expectation Trajectories

We aimed to explore how Big Five personality traits are related to different trajectories of fertility expectations. The largest differences in personality trait levels were found to be between the normative, uncertain, and childfree trajectories, which showed relatively stable expectations compared to the other trajectories. Personality traits might play a significant role in initial fertility expectations and their continuity, whereas experiencing switching can be more related to ongoing life circumstances that require adaptation in the expectations (Heckhausen 1999).

We found the most robust evidence for the link between the trajectories of fertility expectations and agreeableness and extraversion in both men and women. We found that people in the normative class have higher levels of agreeableness and extraversion compared to the uncertain and childfree classes and additionally compared to the postponement class for women. This finding was mostly in line with our expectations and previous research (Avison and Furnham 2015; Berg et al. 2013; Jokela et al. 2011; Jokela 2012; Le Moglie et al. 2015; Lundberg 2009; Pinquart et al. 2008). Extraversion is related to being more energized by social interactions, which may be related to more opportunities to find a partner and pursue a bigger family. In addition, extraversion is related to less ambivalence in the expression of emotions and thus associated with better emotion regulation (Kokkonen and Pulkkinen 2001). This could be related to a more steady and certain expression of expecting to be a parent compared to being unsure about it. Moreover, agreeableness represents warmth, sympathy, and being cooperative. It is related to perceiving parenthood as a positive experience (de Nijs et al. 2014). People with higher levels of agreeableness might make their fertility decisions easier and keep them more certain compared to people with lower levels of agreeableness, which is related to being less cooperative and more selfish (Pinquart et al. 2008).

For the other three traits, we only found differences for men. Lower levels of neuroticism, higher levels of openness, and higher levels of conscientiousness were observed for the men in the normative class compared to the uncertain and childfree classes. The finding for neuroticism supported our expectations and contributed to the previous mixed findings (Avison and Furnham 2015; Berg et al. 2013; Jokela et al. 2011; Jokela 2012; Le Moglie et al. 2015). Interestingly, our finding regarding openness is contradictory to our expectations and to the previous studies that found higher openness is related to fertility postponement and lower fertility in men (Avison and Furnham 2015; Berg et al. 2013; Jokela 2012; Jokela et al. 2011; Le Moglie et al. 2015; Lundberg 2009; van Scheppingen et al. 2016). Our findings might be related to the variance in the fertility concepts in the literature (Philipov and Bernardi 2012). Openness might be related to being open to many experiences in the long term (e.g., becoming a parent one day) but keeping the options open in the short‐term plans (i.e., not wanting to commit to a certain life path too soon). Lastly, our findings regarding conscientiousness are in line with the facets of the trait. For example, being responsible, careful, and planned in life may be related to being steadier and more certain in parenthood decisions. However, different from our expectations, men in the childfree class also had lower conscientiousness levels compared to the normative class whereas they showed steady and certain fertility expectations as well. This finding adds to the mixed literature that found positive (Berg et al. 2013) and no association (Jokela et al. 2011; Jokela 2012) between conscientiousness and fertility behavior in men. Future research is needed to further explain and examine possible mechanisms playing a role between the personality traits and the development of fertility expectations.

Importantly, we found that all Big Five personality traits were related to the fertility expectation classes of men whereas only agreeableness and extraversion were related to the fertility expectation classes of women. This gender difference may be explained by a phenomenon termed as “the motherhood mandate” (Russo 1976). The motherhood mandate refers to the centrality of motherhood for women and that people still expect women to meet this role (Russo 1976; Szekeres et al. 2023). This may be hindering the role of personality characteristics of women on their fertility expectations, unlike men. Future research can explore more on changing norms and personal characteristic on fertility decisions in regard to gender differences.

This study provides a unique contribution by examining the influence of the Big Five personality traits on the trajectory of fertility expectations. While the broader literature on fertility behaviors generally finds that socioeconomic factors (e.g., income, marital status, and education) exert more consistent effects than personality traits (Jokela et al. 2009; Lundberg 2009; Peters 2023; Tavares 2010), psychological attributes may play a stronger role in shaping expectations. To date, research on the development of fertility expectations has largely focused on sociodemographic variables such as marital status, employment, and education (Gemmill 2019; Hayford 2009; Rybińska and Morgan 2019). A notable exception is Buhr and Huinink (2017), who demonstrated that emotional autonomy, a personality trait, contributes to expected family size, yet some structural factors like gender and the influence of friends are more decisive in the decision to give up on having children. In line with this study, our findings underscore the relevance of personality traits in the formation of fertility expectations.

### 4.3 Strengths and Limitations

The strengths of the current study include the fact that the longitudinal design of the study allowed us to observe fertility expectations during the reproductive age period. Using joint latent class modeling that simultaneously analyzes the longitudinal outcome and a time‐to‐event variable (Proust‐Lima et al. 2014), we were able to investigate fertility expectation trajectories for people who became parents and non‐parents. Our findings show that long‐term fertility expectations were associated with becoming a parent in the coming years and therefore deserve more attention in future research. Moreover, we used a biased adjusted 3‐step approach to maximize the accuracy of classification of individuals into the different latent classes. By taking misclassification into account (see Vermunt 2010), we were able to obtain accurate estimates of the relation between identified fertility expectation trajectories and the Big Five personality traits. Lastly, we presented a more complete picture by observing unique trajectories for both men and women and including people who were unsure whether to have a child or not. Future studies should further include more diverse samples, including people of different genders (e.g., transgender, non‐binary). Moreover, replication is needed across countries where freedom of choice and advanced fertility regulation technologies are not as accessible and perceived norms on parenthood are different compared to the Netherlands.

This study has some important limitations. First, even though the average number of waves was almost 4 years, we could observe only parts of the reproductive age period for every person, rather than throughout the whole period. Ideally, future studies can follow fertility expectations of all individuals from an early age across their fertility period. Moreover, the Big Five personality traits were assessed at a single time point, specifically at each participant's first observation. Although personality is often considered relatively stable, research suggests that mean levels of traits can change over time in response to normative developmental processes or life events (Bleidorn et al. 2022; Bühler et al. 2023). Therefore, personality measured at the first observation may not perfectly reflect personality at later time points. This study aimed to examine whether initial personality traits predict fertility expectation trajectories. Given the potentially dynamic nature of personality characteristics, it is particularly noteworthy that we observed meaningful differences between the trajectories despite relying on a single time measure. Future research could build on this by examining how changes in personality co‐occur with changes in fertility decision‐making. Finally, we aimed to present a comprehensive overview of the link between the Big Five personality traits and different fertility expectations trajectories. Future studies can use our study as a starting point to study possible mediators (e.g., having a stable romantic relationship) and life events that may affect the link between personality characteristics and fertility expectations. Furthermore, our innovative modeling approach could be used to study how other individual‐level factors, such as health and well‐being, are related to fertility expectations.

## 5 Conclusion

A relatively stable expectation to become a parent (i.e., the normative trajectory) is the most prevalent trajectory in young adulthood in the Netherlands. However, the majority of our sample deviated from this trajectory by showing different changes in their fertility expectations (i.e., postponement, uncertain, switching, childfree trajectories for both genders, and, additionally, abandoning trajectory for women). This may imply that traditional norms around expecting to be a parent have been challenged. Men and women were remarkably similar in the identified trajectories. One notable gender difference was that a proportion of women abandoned their expectation to become a parent at an earlier age, a class that was not found in men. Individuals in the normative class, regardless of gender, scored higher on agreeableness and extraversion. Men in the normative class showed higher levels of conscientiousness and openness, and lower levels of neuroticism compared to uncertain and childfree classes. More studies are needed to explore underlying mechanisms between personal characteristics and fertility expectations.

## Author Contributions

İlayda Özoruç played the leading role in writing – original draft, formal analysis, and visualization, an equal role in conceptualization, and writing – review and editing, and a supporting role in methodology. Jeroen Vermunt played a leading role in methodology and a supporting role in conceptualization, writing – review and editing, supervision, and formal analysis. Katya Ivanova played an equal role in conceptualization, funding acquisition, and writing – review and editing, and a supporting role in supervision, methodology, and formal analysis. Manon A. van Scheppingen played a leading role in supervision, an equal role in conceptualization, funding acquisition, and writing – review and editing, and a supporting role in methodology and formal analysis.

## Conflicts of Interest

The authors declare no conflicts of interest.

## Supporting information


**Table S1:** jopy70021‐sup‐0001‐TablesS1‐S4.docx.

## Data Availability

The study materials and data can be accessed via the LISS panel website (for Family and Household data and codebook archive, see: https://www.dataarchive.lissdata.nl/study‐units/view/10; for Personality data and codebook archive, see: https://www.dataarchive.lissdata.nl/study‐units/view/14). Note that the LISS Data Archive is free to use for researchers if the data will be used for scientific, policy, or socially relevant (i.e., non‐commercial) research, but researchers need to sign a data user statement confirming their agreement with the rules and conditions beforehand (see for the details: https://www.lissdata.nl/use‐the‐data). The preregistration (registered prior to conducting the main analyses) and analysis scripts used for this article can be accessed via https://osf.io/enbvs/?view_only=c7a474398e3f4fc7b67c3c61de46a4c7. There are four script documents: three of them are for data preparation and descriptive statistics, and one of them is for both the joint latent class model and the biased adjusted 3‐step approach in Latent GOLD.

## Endnotes

^1^
This number slightly deviates from our preregistration. Initially, we excluded participants lacking gender or age information. Because we added the monthly collected background data, we were able to fill some of the missing gender or age values and maximized the number of participants.
