# Supplementary material for: Big Five Personality Traits and Trajectories of Fertility Expectations Across the Reproductive Age Period
Source: J Pers. 2025 Sep 10;94(4):547–62. doi: 10.1111/jopy.70021 (PMC13359319; doi:10.1111/jopy.70021)
Supplement: Supplementary file 1 — Table S1: jopy70021‐sup‐0001‐TablesS1‐S4.docx. [file JOPY-94-547-s001.docx]

**Supplemental Material**

**Table S1**

*Regression Parameters* *for the 5 Class Model for Men*

|  | Term |  | | *Coef* | | *S.E.* | | *z* | | | *p* | | |  |
| --- | --- | --- | --- | --- | --- | --- | --- | --- | --- | --- | --- | --- | --- | --- |
| Expectation(no) | Intercept | class(1) | | -2.30 | | 0.38 | | -5.98 | | | < .001 | | |  |
| Expectation(yes) | Intercept | class(1) | | 1.86 | | 0.21 | | 8.68 | | | < .001 | | |  |
| Expectation(unsure) | Intercept | class(1) | | 0.44 | | 0.23 | | 1.92 | | | .055 | | |  |
| Expectation(no) | B-spline(1) | class(1) | | 1.63 | | 1.06 | | 1.54 | | | .120 | | |  |
| Expectation(yes) | B-spline(1) | class(1) | | 0.33 | | 0.60 | | 0.55 | | | .590 | | |  |
| Expectation(unsure) | B-spline(1) | class(1) | | -1.96 | | 0.66 | | -2.98 | | | .003 | | |  |
| Expectation(no) | B-spline(2) | class(1) | | -7.73 | | 1.29 | | -5.97 | | | < .001 | | |  |
| Expectation(yes) | B-spline(2) | class(1) | | 5.92 | | 1.00 | | 5.90 | | | < .001 | | |  |
| Expectation(unsure) | B-spline(2) | class(1) | | 1.81 | | 0.72 | | 2.51 | | | .012 | | |  |
| Expectation(no) | B-spline(3) | class(1) | | 10.35 | | 1.00 | | 10.31 | | | < .001 | | |  |
| Expectation(yes) | B-spline(3) | class(1) | | -10.34 | | 1.21 | | -8.54 | | | < .001 | | |  |
| Expectation(unsure) | B-spline(3) | class(1) | | -0.02 | | 0.72 | | -0.03 | | | .980 | | |  |
| Expectation(no) | B-spline(4) | class(1) | | 5.94 | | 2.80 | | 2.12 | | | .034 | | |  |
| Expectation(yes) | B-spline(4) | class(1) | | -6.49 | | 5.55 | | -1.17 | | | .240 | | |  |
| Expectation(unsure) | B-spline(4) | class(1) | | 0.55 | | 2.80 | | 0.20 | | | .840 | | |  |
| Parenthood | Intercept | class(1) | | -28.51 | | 9.36 | | -3.05 | | | .002 | | |  |
| Parenthood | B-spline(1) | class(1) | | 29.61 | | 11.80 | | 2.51 | | | .012 | | |  |
| Parenthood | B-spline(2) | class(1) | | 21.70 | | 6.86 | | 3.16 | | | .002 | | |  |
| Parenthood | B-spline(3) | class(1) | | 34.32 | | 12.42 | | 2.76 | | | .006 | | |  |
| Parenthood | B-spline(4) | class(1) | | -62.82 | | 33.22 | | -1.89 | | | .059 | | |  |
| Expectation(no) | Intercept | class(2) | | -2.77 | | 1.49 | | -1.85 | | | .064 | | |  |
| Expectation(yes) | Intercept | class(2) | | 0.36 | | 0.81 | | 0.44 | | | .660 | | |  |
| Expectation(unsure) | Intercept | class(2) | | 2.41 | | 0.74 | | 3.26 | | | .001 | | |  |
| Expectation(no) | B-spline(1) | class(2) | | 0.74 | | 2.62 | | 0.28 | | | .780 | | |  |
| Expectation(yes) | B-spline(1) | class(2) | | -0.26 | | 1.45 | | -0.18 | | | .860 | | |  |
| Expectation(unsure) | B-spline(1) | class(2) | | -0.49 | | 1.35 | | -0.36 | | | .720 | | |  |
| Expectation(no) | B-spline(2) | class(2) | | -0.09 | | 3.46 | | -0.03 | | | .980 | | |  |
| Expectation(yes) | B-spline(2) | class(2) | | 4.47 | | 1.83 | | 2.44 | | | .015 | | |  |
| Expectation(unsure) | B-spline(2) | class(2) | | -4.38 | | 1.86 | | -2.35 | | | .019 | | |  |
| Expectation(no) | B-spline(3) | class(2) | | -3.44 | | 3.66 | | -0.94 | | | .350 | | |  |
| Expectation(yes) | B-spline(3) | class(2) | | 2.28 | | 2.01 | | 1.13 | | | .260 | | |  |
| Expectation(unsure) | B-spline(3) | class(2) | | 1.16 | | 1.92 | | 0.60 | | | .550 | | |  |
| Expectation(no) | B-spline(4) | class(2) | | 13.72 | | 4.38 | | 3.13 | | | .002 | | |  |
| Expectation(yes) | B-spline(4) | class(2) | | -6.81 | | 2.58 | | -2.64 | | | .008 | | |  |
| Expectation(unsure) | B-spline(4) | class(2) | | -6.91 | | 2.54 | | -2.71 | | | .007 | | |  |
| Parenthood | Intercept | class(2) | | -6.91 | | 1.70 | | -4.07 | | | < .001 | | |  |
| Parenthood | B-spline(1) | class(2) | | 2.73 | | 2.66 | | 1.03 | | | .300 | | |  |
| Parenthood | B-spline(2) | class(2) | | 5.01 | | 1.58 | | 3.16 | | | .002 | | |  |
| Parenthood | B-spline(3) | class(2) | | 6.29 | | 2.10 | | 3.00 | | | .003 | | |  |
| Parenthood | B-spline(4) | class(2) | | 3.47 | | 1.78 | | 1.95 | | | .052 | | |  |
| Expectation(no) | Intercept | class(3) | | -2.11 | | 0.57 | | -3.72 | | | < .001 | | |  |
| Expectation(yes) | Intercept | class(3) | | 1.40 | | 0.35 | | 4.02 | | | < .001 | | |  |
| Expectation(unsure) | Intercept | class(3) | | 0.71 | | 0.33 | | 2.15 | | | .032 | | |  |
| Expectation(no) | B-spline(1) | class(3) | | 1.22 | | 1.02 | | 1.19 | | | .230 | | |  |
| Expectation(yes) | B-spline(1) | class(3) | | -2.14 | | 0.67 | | -3.18 | | | .002 | | |  |
| Expectation(unsure) | B-spline(1) | class(3) | | 0.92 | | 0.59 | | 1.54 | | | .120 | | |  |
| Expectation(no) | B-spline(2) | class(3) | | 0.15 | | 0.84 | | 0.18 | | | .860 | | |  |
| Expectation(yes) | B-spline(2) | class(3) | | -0.97 | | 0.67 | | -1.44 | | | .150 | | |  |
| Expectation(unsure) | B-spline(2) | class(3) | | 0.82 | | 0.49 | | 1.67 | | | .096 | | |  |
| Expectation(no) | B-spline(3) | class(3) | | 0.54 | | 0.87 | | 0.63 | | | .530 | | |  |
| Expectation(yes) | B-spline(3) | class(3) | | -1.88 | | 0.73 | | -2.60 | | | .009 | | |  |
| Expectation(unsure) | B-spline(3) | class(3) | | 1.34 | | 0.55 | | 2.43 | | | .015 | | |  |
| Expectation(no) | B-spline(4) | class(3) | | 3.22 | | 0.73 | | 4.44 | | | < .001 | | |  |
| Expectation(yes) | B-spline(4) | class(3) | | -4.18 | | 0.89 | | -4.71 | | | < .001 | | |  |
| Expectation(unsure) | B-spline(4) | class(3) | | 0.97 | | 0.52 | | 1.85 | | | .064 | | |  |
| Parenthood | Intercept | class(3) | | -40.24 | | 50.55 | | -0.80 | | | .430 | | |  |
| Parenthood | B-spline(1) | class(3) | | 39.44 | | 54.42 | | 0.72 | | | .470 | | |  |
| Parenthood | B-spline(2) | class(3) | | 34.81 | | 48.23 | | 0.72 | | | .470 | | |  |
| Parenthood | B-spline(3) | class(3) | | 37.46 | | 51.43 | | 0.73 | | | .470 | | |  |
| Parenthood | B-spline(4) | class(3) | | 35.48 | | 50.29 | | 0.71 | | | .480 | | |  |
| Expectation(no) | Intercept | | class(4) | | -9.90 | | 5.84 | | -1.70 | | | .090 | | |
| Expectation(yes) | Intercept | | class(4) | | 7.73 | | 3.13 | | 2.47 | | | .014 | | |
| Expectation(unsure) | Intercept | | class(4) | | 2.17 | | 2.97 | | 0.73 | | | .470 | | |
| Expectation(no) | B-spline(1) | | class(4) | | 7.91 | | 6.84 | | 1.16 | | | .250 | | |
| Expectation(yes) | B-spline(1) | | class(4) | | -5.83 | | 3.86 | | -1.51 | | | .130 | | |
| Expectation(unsure) | B-spline(1) | | class(4) | | -2.08 | | 3.50 | | -0.59 | | | .550 | | |
| Expectation(no) | B-spline(2) | | class(4) | | 10.92 | | 5.27 | | 2.07 | | | .038 | | |
| Expectation(yes) | B-spline(2) | | class(4) | | -10.42 | | 2.78 | | -3.75 | | | < .001 | | |
| Expectation(unsure) | B-spline(2) | | class(4) | | -0.51 | | 2.70 | | -0.19 | | | .850 | | |
| Expectation(no) | B-spline(3) | | class(4) | | 11.79 | | 6.14 | | 1.92 | | | .055 | | |
| Expectation(yes) | B-spline(3) | | class(4) | | -9.36 | | 3.44 | | -2.72 | | | .007 | | |
| Expectation(unsure) | B-spline(3) | | class(4) | | -2.43 | | 3.17 | | -0.76 | | | .440 | | |
| Expectation(no) | B-spline(4) | | class(4) | | 9.13 | | 5.74 | | 1.59 | | | .110 | | |
| Expectation(yes) | B-spline(4) | | class(4) | | -7.80 | | 3.09 | | -2.53 | | | .012 | | |
| Expectation(unsure) | B-spline(4) | | class(4) | | -1.33 | | 2.93 | | -0.45 | | | .650 | | |
| Parenthood | Intercept | | class(4) | | -7.17 | | 2.31 | | -3.10 | | | .002 | | |
| Parenthood | B-spline(1) | | class(4) | | 5.03 | | 4.35 | | 1.16 | | | .250 | | |
| Parenthood | B-spline(2) | | class(4) | | -0.97 | | 3.35 | | -0.29 | | | .770 | | |
| Parenthood | B-spline(3) | | class(4) | | 7.51 | | 3.62 | | 2.08 | | | .038 | | |
| Parenthood | B-spline(4) | | class(4) | | 1.54 | | 3.10 | | 0.50 | | | .620 | | |
| Expectation(no) | Intercept | | class(5) | | 2.12 | | 0.89 | | | 2.39 | | | .017 | |
| Expectation(yes) | Intercept | | class(5) | | -4.20 | | 1.76 | | | -2.39 | | | .017 | |
| Expectation(unsure) | Intercept | | class(5) | | 2.08 | | 0.91 | | | 2.29 | | | .022 | |
| Expectation(no) | B-spline(1) | | class(5) | | -3.52 | | 2.16 | | | -1.63 | | | .100 | |
| Expectation(yes) | B-spline(1) | | class(5) | | 6.45 | | 4.30 | | | 1.50 | | | .130 | |
| Expectation(unsure) | B-spline(1) | | class(5) | | -2.93 | | 2.28 | | | -1.28 | | | .200 | |
| Expectation(no) | B-spline(2) | | class(5) | | 5.53 | | 3.01 | | | 1.84 | | | .066 | |
| Expectation(yes) | B-spline(2) | | class(5) | | -5.53 | | 5.86 | | | -0.94 | | | .350 | |
| Expectation(unsure) | B-spline(2) | | class(5) | | 0.00 | | 3.34 | | | 0.00 | | | 1 | |
| Expectation(no) | B-spline(3) | | class(5) | | -1.61 | | 5.75 | | | -0.28 | | | .780 | |
| Expectation(yes) | B-spline(3) | | class(5) | | 0.99 | | 11.24 | | | 0.09 | | | .930 | |
| Expectation(unsure) | B-spline(3) | | class(5) | | 0.62 | | 6.32 | | | 0.10 | | | .920 | |
| Expectation(no) | B-spline(4) | | class(5) | | 55.39 | | 34.46 | | | 1.61 | | | .110 | |
| Expectation(yes) | B-spline(4) | | class(5) | | 50.26 | | 36.86 | | | 1.36 | | | .170 | |
| Expectation(unsure) | B-spline(4) | | class(5) | | -105.65 | | 67.54 | | | -1.56 | | | .120 | |
| Parenthood | Intercept | | class(5) | | -10.48 | | 32.82 | | | -0.32 | | | .750 | |
| Parenthood | B-spline(1) | | class(5) | | -262.20 | | 317.95 | | | -0.82 | | | .410 | |
| Parenthood | B-spline(2) | | class(5) | | 197.51 | | 225.18 | | | 0.88 | | | .380 | |
| Parenthood | B-spline(3) | | class(5) | | -123.30 | | 156.91 | | | -0.79 | | | .430 | |
| Parenthood | B-spline(4) | | class(5) | | 0.24 | | 50.06 | | | 0.00 | | | 1 | |

*Note.* Coefficients show the class-specific B-spline coefficients, it is advised to use the graphs of the probabilities (Figure 1 in the main text) for an easier interpretation.

**Table S2**

*Model Comparisons With Increased Knots for 1-6 Class Models for Men*

| Model | Number of classes | BIC | AIC | AIC3 | VLMR | Entropy R² |
| --- | --- | --- | --- | --- | --- | --- |
| 1knot | 1-class | 17816.49 | 17729.09 | 17744.09 |  | 1 |
| 1knot | 2-class | 15014.93 | 14834.29 | 14865.29 | 2926.80  *p* < .001 | 0.688 |
| 1knot | 3-class | 14429.82 | 14155.96 | 14202.96 | 710.34  *p* < .001 | 0.637 |
| 1knot | 4-class | 14252.71 | 13885.62 | 13948.620 | 302.34 *p* < .001 | 0.533 |
| 1knot | 5-class | 14162.78 | 13702.46 | 13781.46 | 215.15 *p* < .001 | 0.503 |
| 1knot | 6-class | 14188.59 | 13635.04 | 13730.04 | 99.43  *p* < .001 | 0.460 |
| 2knots | 1-class | 17841.16 | 17736.28 | 17754.28 |  | 1 |
| 2knots | 2-class | 15054.73 | 14839.13 | 14876.13 | 2935.14 *p* < .001 | 0.689 |
| 2knots | 3-class | 14487.40 | 14161.10 | 14217.10 | 716.04  *p* < .001 | 0.639 |
| 2knots | 4-class | 14329.48 | 13892.47 | 13967.47 | 306.62 *p* < .001 | 0.533 |
| 2knots | 5-class | 14266.14 | 13718.42 | 13812.42 | 212.05 | 0.498 |
| 2knots | 6-class | 14309.01 | 13650.58 | 13763.58 | 105.84  *p* = .001 | 0.4697 |
| 3knots | 1-class | 17857.37 | 17735.01 | 17756.01 |  | 1 |
| 3knots | 2-class | 15098.38 | 14847.83 | 14890.83 | 2931.18 *p* < .001 | 0.688 |
| 3knots | 3-class | 14536.13 | 14157.38 | 14222.38 | 734.44 *p* < .001 | 0.639 |
| 3knots | 4-class | 14403.95 | 13897.01 | 13984.01 | 304.37 *p* < .001 | 0.539 |
| 3knots | 5-class | 14354.33 | 13719.20 | 13828.20 | 221.81 *p* < .001 | 0.506 |
| 3knots | 6-class | 14421.69 | 13658.38 | 13789.38 | 104.82  *p* = .013 | 0.469 |

*Note.* AIC = Akaike information criterion; BIC = Bayesian information criterion; VLMR = Vuong- Lo-Mendel-Rubin test

**Table S3**

*Regression Parameters* *for the 6 Class Model for Women*

|  | Term |  | *Coef* | *S.E.* | *z* | *p* |
| --- | --- | --- | --- | --- | --- | --- |
| Expectation(no) | Intercept | class(1) | -7.57 | 2.76 | -2.74 | .006 |
| Expectation(yes) | Intercept | class(1) | 5.12 | 1.40 | 3.65 | < .001 |
| Expectation(unsure) | Intercept | class(1) | 2.45 | 1.49 | 1.64 | .100 |
| Expectation(no) | B-spline(1) | class(1) | 13.25 | 5.15 | 2.57 | .010 |
| Expectation(yes) | B-spline(1) | class(1) | -5.74 | 2.66 | -2.16 | .031 |
| Expectation(unsure) | B-spline(1) | class(1) | -7.51 | 2.77 | -2.71 | .007 |
| Expectation(no) | B-spline(2) | class(1) | -12.34 | 4.16 | -2.97 | .003 |
| Expectation(yes) | B-spline(2) | class(1) | 10.29 | 2.35 | 4.38 | < .001 |
| Expectation(unsure) | B-spline(2) | class(1) | 2.05 | 2.38 | 0.86 | .390 |
| Expectation(no) | B-spline(3) | class(1) | 10.59 | 3.63 | 2.92 | .004 |
| Expectation(yes) | B-spline(3) | class(1) | -8.77 | 2.02 | -4.35 | < .001 |
| Expectation(unsure) | B-spline(3) | class(1) | -1.82 | 1.94 | -0.94 | .350 |
| Expectation(no) | B-spline(4) | class(1) | 10.14 | 2.67 | 3.80 | < .001 |
| Expectation(yes) | B-spline(4) | class(1) | -7.37 | 1.59 | -4.65 | < .001 |
| Expectation(unsure) | B-spline(4) | class(1) | -2.77 | 1.57 | -1.76 | .078 |
| Parenthood | Intercept | class(1) | -29.49 | 13.33 | -2.21 | .027 |
| Parenthood | B-spline(1) | class(1) | 26.43 | 14.71 | 1.80 | .072 |
| Parenthood | B-spline(2) | class(1) | 29.39 | 12.38 | 2.37 | .018 |
| Parenthood | B-spline(3) | class(1) | 27.44 | 13.87 | 1.98 | .048 |
| Parenthood | B-spline(4) | class(1) | 24.61 | 13.09 | 1.88 | .060 |
| Expectation(no) | Intercept | class(2) | -120.77 | 158.16 | -0.76 | .450 |
| Expectation(yes) | Intercept | class(2) | 61.16 | 79.13 | 0.77 | .440 |
| Expectation(unsure) | Intercept | class(2) | 59.61 | 79.04 | 0.75 | .450 |
| Expectation(no) | B-spline(1) | class(2) | 120.98 | 164.81 | 0.73 | .460 |
| Expectation(yes) | B-spline(1) | class(2) | -57.77 | 82.30 | -0.70 | .480 |
| Expectation(unsure) | B-spline(1) | class(2) | -63.21 | 82.52 | -0.77 | .440 |
| Expectation(no) | B-spline(2) | class(2) | 116.75 | 153.65 | 0.76 | .450 |
| Expectation(yes) | B-spline(2) | class(2) | -61.76 | 77.29 | -0.80 | .420 |
| Expectation(unsure) | B-spline(2) | class(2) | -54.99 | 76.40 | -0.72 | .470 |
| Expectation(no) | B-spline(3) | class(2) | 124.63 | 160.88 | 0.77 | .440 |
| Expectation(yes) | B-spline(3) | class(2) | -64.20 | 79.74 | -0.81 | .420 |
| Expectation(unsure) | B-spline(3) | class(2) | -60.44 | 81.18 | -0.74 | .460 |
| Expectation(no) | B-spline(4) | class(2) | 135.92 | 152.68 | 0.89 | .370 |
| Expectation(yes) | B-spline(4) | class(2) | -55.21 | 82.44 | -0.67 | .500 |
| Expectation(unsure) | B-spline(4) | class(2) | -80.71 | 72.69 | -1.11 | .270 |
| Parenthood | Intercept | class(2) | -5.13 | 1.09 | -4.70 | < .001 |
| Parenthood | B-spline(1) | class(2) | 2.61 | 1.93 | 1.36 | .170 |
| Parenthood | B-spline(2) | class(2) | 2.75 | 2.08 | 1.33 | .180 |
| Parenthood | B-spline(3) | class(2) | 1.16 | 2.24 | 0.52 | .600 |
| Parenthood | B-spline(4) | class(2) | 1.46 | 1.88 | 0.78 | .440 |
| Expectation(no) | Intercept | class(3) | -2.37 | 0.84 | -2.81 | .005 |
| Expectation(yes) | Intercept | class(3) | 0.70 | 0.51 | 1.38 | .170 |
| Expectation(unsure) | Intercept | class(3) | 1.67 | 0.42 | 3.98 | < .001 |
| Expectation(no) | B-spline(1) | class(3) | 1.91 | 2.16 | 0.89 | .380 |
| Expectation(yes) | B-spline(1) | class(3) | -0.84 | 1.30 | -0.65 | .520 |
| Expectation(unsure) | B-spline(1) | class(3) | -1.07 | 1.07 | -1.00 | .320 |
| Expectation(no) | B-spline(2) | class(3) | -8.48 | 2.12 | -4.00 | < .001 |
| Expectation(yes) | B-spline(2) | class(3) | 9.64 | 1.53 | 6.30 | < .001 |
| Expectation(unsure) | B-spline(2) | class(3) | -1.16 | 1.19 | -0.97 | .330 |
| Expectation(no) | B-spline(3) | class(3) | 8.66 | 1.72 | 5.03 | < .001 |
| Expectation(yes) | B-spline(3) | class(3) | -10.96 | 1.82 | -6.04 | < .001 |
| Expectation(unsure) | B-spline(3) | class(3) | 2.30 | 1.13 | 2.04 | .041 |
| Expectation(no) | B-spline(4) | class(3) | 6.98 | 3.90 | 1.79 | .074 |
| Expectation(yes) | B-spline(4) | class(3) | -4.47 | 7.56 | -0.59 | .550 |
| Expectation(unsure) | B-spline(4) | class(3) | -2.51 | 3.90 | -0.64 | .520 |
| Parenthood | Intercept | class(3) | -7.71 | 5.20 | -1.48 | .140 |
| Parenthood | B-spline(1) | class(3) | 5.56 | 8.45 | 0.66 | .510 |
| Parenthood | B-spline(2) | class(3) | -1.81 | 5.05 | -0.36 | .720 |
| Parenthood | B-spline(3) | class(3) | 21.54 | 13.54 | 1.59 | .110 |
| Parenthood | B-spline(4) | class(3) | -527.53 | 327.86 | -1.61 | .110 |
| Expectation(no) | Intercept | class(4) | -2.62 | 0.94 | -2.80 | .005 |
| Expectation(yes) | Intercept | class(4) | 1.02 | 0.52 | 1.97 | .049 |
| Expectation(unsure) | Intercept | class(4) | 1.60 | 0.48 | 3.36 | .001 |
| Expectation(no) | B-spline(1) | class(4) | 0.31 | 1.54 | 0.20 | .840 |
| Expectation(yes) | B-spline(1) | class(4) | -1.55 | 0.99 | -1.57 | .120 |
| Expectation(unsure) | B-spline(1) | class(4) | 1.25 | 0.83 | 1.50 | .130 |
| Expectation(no) | B-spline(2) | class(4) | 3.51 | 1.19 | 2.96 | .003 |
| Expectation(yes) | B-spline(2) | class(4) | -2.34 | 1.02 | -2.29 | .022 |
| Expectation(unsure) | B-spline(2) | class(4) | -1.17 | 0.69 | -1.69 | .092 |
| Expectation(no) | B-spline(3) | class(4) | 0.04 | 1.38 | 0.03 | .970 |
| Expectation(yes) | B-spline(3) | class(4) | -0.41 | 1.18 | -0.35 | .730 |
| Expectation(unsure) | B-spline(3) | class(4) | 0.37 | 0.78 | 0.47 | .640 |
| Expectation(no) | B-spline(4) | class(4) | 4.96 | 1.34 | 3.69 | < .001 |
| Expectation(yes) | B-spline(4) | class(4) | -5.06 | 1.86 | -2.72 | .007 |
| Expectation(unsure) | B-spline(4) | class(4) | 0.10 | 1.01 | 0.10 | .920 |
| Parenthood | Intercept | class(4) | -13.43 | 9.56 | -1.40 | .160 |
| Parenthood | B-spline(1) | class(4) | 11.14 | 12.24 | 0.91 | .360 |
| Parenthood | B-spline(2) | class(4) | 7.05 | 8.03 | 0.88 | .380 |
| Parenthood | B-spline(3) | class(4) | 12.91 | 10.42 | 1.24 | .220 |
| Parenthood | B-spline(4) | class(4) | 8.38 | 9.28 | 0.90 | .370 |
| Expectation(no) | Intercept | class(5) | -2.97 | 2.75 | -1.08 | .280 |
| Expectation(yes) | Intercept | class(5) | 4.15 | 1.61 | 2.58 | .010 |
| Expectation(unsure) | Intercept | class(5) | -1.17 | 1.52 | -0.77 | .440 |
| Expectation(no) | B-spline(1) | class(5) | -2.98 | 4.33 | -0.69 | .490 |
| Expectation(yes) | B-spline(1) | class(5) | -2.28 | 2.08 | -1.10 | .270 |
| Expectation(unsure) | B-spline(1) | class(5) | 5.26 | 2.81 | 1.87 | .061 |
| Expectation(no) | B-spline(2) | class(5) | 6.00 | 5.24 | 1.14 | .250 |
| Expectation(yes) | B-spline(2) | class(5) | -5.13 | 2.76 | -1.86 | .063 |
| Expectation(unsure) | B-spline(2) | class(5) | -0.86 | 2.61 | -0.33 | .740 |
| Expectation(no) | B-spline(3) | class(5) | -2.80 | 4.60 | -0.61 | .540 |
| Expectation(yes) | B-spline(3) | class(5) | 1.29 | 2.20 | 0.59 | .560 |
| Expectation(unsure) | B-spline(3) | class(5) | 1.51 | 3.04 | 0.50 | .620 |
| Expectation(no) | B-spline(4) | class(5) | 4.05 | 2.88 | 1.41 | .160 |
| Expectation(yes) | B-spline(4) | class(5) | -7.15 | 1.81 | -3.96 | < .001 |
| Expectation(unsure) | B-spline(4) | class(5) | 3.10 | 1.60 | 1.94 | .052 |
| Parenthood | Intercept | class(5) | -15.37 | 16.17 | -0.95 | .340 |
| Parenthood | B-spline(1) | class(5) | 11.40 | 18.59 | 0.61 | .540 |
| Parenthood | B-spline(2) | class(5) | 11.50 | 15.01 | 0.77 | .440 |
| Parenthood | B-spline(3) | class(5) | 15.41 | 16.68 | 0.92 | .360 |
| Parenthood | B-spline(4) | class(5) | 12.60 | 16.02 | 0.79 | .430 |
| Expectation(no) | Intercept | class(6) | 0.17 | 0.20 | 0.88 | .380 |
| Expectation(yes) | Intercept | class(6) | -0.15 | 0.26 | -0.58 | .560 |
| Expectation(unsure) | Intercept | class(6) | -0.02 | 0.23 | -0.11 | .920 |
| Expectation(no) | B-spline(1) | class(6) | 1.22 | 0.59 | 2.06 | .039 |
| Expectation(yes) | B-spline(1) | class(6) | -1.51 | 0.93 | -1.63 | .100 |
| Expectation(unsure) | B-spline(1) | class(6) | 0.29 | 0.67 | 0.44 | .660 |
| Expectation(no) | B-spline(2) | class(6) | -2.16 | 1.29 | -1.67 | .094 |
| Expectation(yes) | B-spline(2) | class(6) | 3.97 | 2.42 | 1.64 | .100 |
| Expectation(unsure) | B-spline(2) | class(6) | -1.81 | 1.40 | -1.29 | .200 |
| Expectation(no) | B-spline(3) | class(6) | 10.33 | 3.41 | 3.03 | .003 |
| Expectation(yes) | B-spline(3) | class(6) | -15.96 | 6.65 | -2.40 | .016 |
| Expectation(unsure) | B-spline(3) | class(6) | 5.63 | 3.49 | 1.61 | .110 |
| Expectation(no) | B-spline(4) | class(6) | 4.71 | 3.25 | 1.45 | .150 |
| Expectation(yes) | B-spline(4) | class(6) | -2.19 | 5.88 | -0.37 | .710 |
| Expectation(unsure) | B-spline(4) | class(6) | -2.52 | 4.19 | -0.60 | .550 |
| Parenthood | Intercept | class(6) | -10.84 | 32.84 | -0.33 | .740 |
| Parenthood | B-spline(1) | class(6) | -246.59 | 269.53 | -0.91 | .360 |
| Parenthood | B-spline(2) | class(6) | 174.83 | 180.43 | 0.97 | .330 |
| Parenthood | B-spline(3) | class(6) | -95.96 | 117.18 | -0.82 | .410 |
| Parenthood | B-spline(4) | class(6) | 8.55 | 32.86 | 0.26 | .790 |

*Note.* Coefficients show the class-specific B-spline coefficients, it is advised to use the graphs of the probabilities (Figure 2 in the main text) for an easier interpretation.

**Table S4**

*Model Comparisons With Increased Knots for 1-7 Class Models for Women*

| Model | Number of classes | BIC | AIC | AIC3 | VLMR | Entropy R² |
| --- | --- | --- | --- | --- | --- | --- |
| 1knot | 1-class | 18160.28 | 18071.64 | 18086.64 |  | 1 |
| 1knot | 2-class | 15356.11 | 15172.91 | 15203.91 | 2930.73  *p* < .001 | 0.683 |
| 1knot | 3-class | 14832.06 | 14554.29 | 14601.29 | 650.61  *p* < .001 | 0.632 |
| 1knot | 4-class | 14681.29 | 14308.97 | 14371.97 | 277.33  *p* < .001 | 0.552 |
| 1knot | 5-class | 14622.92 | 14156.05 | 14235.05 | 184.92  *p* < .001 | 0.511 |
| 1knot | 6-class | 14614.43 | 14052.99 | 14147.99 | 135.05 *p* < .001 | 0.465 |
| 1knot | 7-class | 14651.93 | 13995.94 | 14106.94 | 89.05 *p* < .001 | 0.459 |
| 2knots | 1-class | 18180.19 | 18073.81 | 18091.81 |  | 1 |
| 2knots | 2-class | 15395.83 | 15177.16 | 15214.16 | 2934.64 *p* < .001 | 0.68 |
| 2knots | 3-class | 14898.72 | 14567.76 | 14623.76 | 647.40 *p* < .001 | 0.633 |
| 2knots | 4-class | 14741.47 | 14298.23 | 14373.23 | 307.53  *p* < .001 | 0.555 |
| 2knots | 5-class | 14726.28 | 14170.75 | 14264.75 | 165.48  *p* < .001 | 0.514 |
| 2knots | 6-class | 14733.08 | 14065.27 | 14178.27 | 143.49  *p* < .001 | 0.465 |
| 2knots | 7-class | 14788.67 | 14008.57 | 14140.57 | 94.69 *p* = .002 | 0.491 |
| 3knots | 1-class | 18198.34 | 18074.24 | 18095.24 |  | 1 |
| 3knots | 2-class | 15414.46 | 15160.34 | 15203.34 | 2957.90  *p* < .001 | 0.687 |
| 3knots | 3-class | 14939.31 | 14555.17 | 14620.17 | 649.16  *p* < .001 | 0.635 |
| 3knots | 4-class | 14828.07 | 14313.92 | 14400.92 | 285.26  *p* < .001 | 0.558 |
| 3knots | 5-class | 14821.72 | 14177.55 | 14286.55 | 180.36 *p* < .001 | 0.512 |
| 3knots | 6-class | 14851.12 | 14076.93 | 14207.93 | 144.62  *p* < .001 | 0.476 |
| 3 knots | 7-class | 14909.53 | 14005.33 | 14158.33 | 115.60  *p* < .001 | 0.472 |

*Note.* AIC = Akaike information criterion; BIC = Bayesian information criterion
